# Genomic mapping of cAMP receptor protein (CRP^Mt^) in *Mycobacterium tuberculosis*: relation to transcriptional start sites and the role of CRP^Mt^ as a transcription factor

**DOI:** 10.1093/nar/gku548

**Published:** 2014-06-21

**Authors:** Christina Kahramanoglou, Teresa Cortes, Nishad Matange, Debbie M. Hunt, Sandhya S. Visweswariah, Douglas B. Young, Roger S. Buxton

**Affiliations:** 1 Division of Mycobacterial Research, MRC National Institute for Medical Research, Mill Hill, London, NW7 1AA, UK; 2 Department of Molecular Reproduction, Development and Genetics, Indian Institute of Science, Bangalore 560012, India; 3 Centre for Molecular Bacteriology and Infection, Imperial College London, SW7 2AZ, UK

## Abstract

Chromatin immunoprecipitation identified 191 binding sites of *Mycobacterium tuberculosis* cAMP receptor protein (CRP^Mt^) at endogenous expression levels using a specific α-CRP^Mt^ antibody. Under these native conditions an equal distribution between intragenic and intergenic locations was observed. CRP^Mt^ binding overlapped a palindromic consensus sequence. Analysis by RNA sequencing revealed widespread changes in transcriptional profile in a mutant strain lacking CRP^Mt^ during exponential growth, and in response to nutrient starvation. Differential expression of genes with a CRP^Mt^-binding site represented only a minor portion of this transcriptional reprogramming with ∼19% of those representing transcriptional regulators potentially controlled by CRP^Mt^. The subset of genes that are differentially expressed in the deletion mutant under both culture conditions conformed to a pattern resembling canonical CRP regulation in *Escherichia coli*, with binding close to the transcriptional start site associated with repression and upstream binding with activation. CRP^Mt^ can function as a classical transcription factor in *M. tuberculosis*, though this occurs at only a subset of CRP^Mt^-binding sites.

## INTRODUCTION

The success of *Mycobacterium tuberculosis* as one of the most deadly human pathogens depends on its ability to adapt to diverse intracellular and extracellular environments encountered during infection, persistence and transmission [reviewed in (1)]. This is mediated in part by an extensive repertoire of transcriptional regulators, including alternative sigma factors, two-component signal transduction proteins and serine–threonine protein kinase sensors (2). Defining the scope of individual regulators and their participation in integrated regulatory networks generates insights into the *in vivo* physiology of *M. tuberculosis* that will assist in the selection of optimal treatment strategies (3). A combination of chromatin immunoprecipitation (ChIP) with sequence-based transcriptional profiling provides a powerful approach to this goal. Whilst some mycobacterial transcription factors display a restricted profile of binding to a limited set of regulated promoters (4–6), a recent study of EspR revealed a much broader profile resembling that of nucleoid-associated proteins (NAPs) involved in structural organization of the chromosome (7,8). High-throughput ChIP experiments based on overexpressed transcription factors in *M. tuberculosis* systematically detect a wide repertoire of low affinity binding sites, however, suggesting that there may be no clear-cut distinction between proteins with localized and generalized binding profiles (9). In the present study, we set out to define the genomic binding profile of cAMP (cyclic adenosine monophosphate) receptor protein (CRP^Mt^) of *M. tuberculosis* with a particular emphasis on the localization of CRP^Mt^-binding sites relative to transcription start sites (TSSs).

CRP is one of the best-characterized transcription factors in the model organism, *Escherichia coli*, with a role in regulation of around 200 transcriptional units. CRP binds to a palindromic sequence (TGTGAN_6_TCACA) in the promoters of target genes and, depending on the distance between the binding site and the transcriptional start site, enhances or restricts recruitment of ribonucleic acid (RNA) polymerase [reviewed in (10,11)]. In addition to binding to target promoters, ChIP-chip analysis of *E. coli* CRP uncovered an extensive background pattern of low affinity sites suggesting that CRP may have an additional role as a more general chromosomal organizer (12). A key feature of *E. coli* CRP is that its affinity for deoxyribonucleic acid (DNA) is strongly dependent on binding of the cAMP ligand, allowing it to play a central role in the global coordination of transcriptional reprogramming required for optimal utilization of different carbon substrates.

The corresponding *M. tuberculosis* CRP^Mt^, encoded by Rv3676, recognizes a similar binding motif and has been shown to regulate transcription of several promoters—for example, the *serC* promoter (13), the *rpfA* promoter (14), the *whiB1* promoter (15,16) and the *frdA* promoter (17). Computational approaches and *in vitro* DNA-binding studies suggest that *M. tuberculosis* CRP^Mt^ has multiple targets and is likely to share the global profile of the *E. coli* homologue (18–20). A significant difference between the two proteins is that cAMP binds to *M. tuberculosis* CRP^Mt^ with weak affinity and has less effect on its binding to DNA [(21), for review see (22)]. This may reflect differences in the abundance and regulation of cAMP in mycobacteria (23,24). In contrast to the single enzyme in *E. coli*, *M. tuberculosis* has 17 genes encoding adenylate cyclases (25,26), and the dynamics of cAMP synthesis and secretion are proposed to play an important role during infection [(27), for review see (23)]. Consistent with a role in pathogenesis, deletion of the *crp* gene results in significant impairment of *in vitro* growth of *M. tuberculosis* and attenuates virulence in a mouse model (14). We anticipated that comprehensive mapping of the binding profile of *M. tuberculosis* CRP^Mt^ would assist in characterization of this global regulator and contribute more broadly to a fundamental understanding of gene regulation in mycobacteria.

## MATERIALS AND METHODS

### Bacterial strains, plasmids and growth conditions

The strains used were *E. coli* strain DH5α, for all plasmid construction, and *M. tuberculosis* strains H37Rv (wild type) and *M. tuberculosis Δcrp*, an H37Rv mutant in which *Rv3676* has been deleted (14). *E. coli* cultures were grown in Luria-Bertani broth and agar (15 g/l). Where needed, ampicillin and kanamycin were used at final concentrations of 100 and 50 μg/ml, respectively. *M. tuberculosis* cultures were grown in Dubos broth containing 0.05% (v/v) Tween, Middlebrook 7H9 broth or Middlebrook 7H11 agar and supplemented with 0.5% (v/v) glycerol and 4% albumin. To monitor the response to nutrient starvation (28), *M. tuberculosis* was grown to mid-exponential phase (OD_600_ 0.6), the cells pelleted and washed twice in phosphate buffered saline (PBS) supplemented with 0.025% tyloxapol. Cells were then resuspended in 100 ml PBS plus tyloxapol and incubated without shaking at 37 °C.

### Chromatin immunoprecipitation

ChIP was performed as previously described (29) with some modifications to the protocol. Rv3676 protein tagged with hexa-His at the N-terminus was purified in *E. coli* as described previously (30) and used to produce CRP^Mt^-specific polyclonal antibodies in rabbits. The primary dose (300-μg protein) was administered subcutaneously in Freund's complete adjuvant, followed by two booster injections in Freund's incomplete adjuvant after 14 and 30 days. Sera were prepared and then stored at −20°C until required. Immunoglobulin G (IgG) was purified from the serum using T-Gel purification kit (Pierce) as per manufacturer's instructions. Purified IgG was used for ChIP-seq analysis. Rv3597c antibody was raised by Cambridge Research Biochemicals, using a peptide antigen.

*M. tuberculosis* H37Rv (wild type) and *M. tuberculosis Δcrp* cells were grown in roller bottles to mid-exponential phase (OD_600_ 0.6) and formaldehyde was added to a final concentration of 1%. After 10 min of incubation, glycine was added to a final concentration of 0.5 M to quench the reaction and incubated for a further 5 min. Cross-linked cells were harvested by centrifugation and washed twice with ice-cold Tris-buffered saline (TBS, pH 7.5). Cell pellets were resuspended in 4-ml immunoprecipitation (IP) buffer [50-mM HEPES-KOH (pH 7.5), 150-mM NaCl, 1-mM ethylenediaminetetraacetic acid (EDTA), 1% Triton X-100, 0.1% (w/v) sodium deoxycholate, 0.1% sodium dodecyl sulphate (SDS), 0.1-mg/ml RNase A and one protease inhibitor cocktail tablet (Roche)]. Cells were lysed and the DNA sheared to an average size of ∼300 base pairs (bp) using a Bioruptor (Diagenode) with 40 cycles of 30-s on/off at high setting. Insoluble matter was removed by centrifugation for 10 min at 4°C, and the supernatant was split into two 1.8-ml aliquots. The remaining ∼400 μl was kept to check the size of the DNA fragments and for sequencing to remove any sequencing bias (input).

Each 1.8-ml aliquot was incubated with 20-μl Protein A/G UltraLink Resin (Pierce) on a rotary shaker for 45 min at room temperature to eliminate complexes bound to the resin non-specifically. The supernatant was then removed and incubated with either no antibody (mock-IP), specific α-CRP^Mt^ antibody raised against purified CRP^Mt^ (Rv3676) protein (Supplementary Figure S1) and 50-μl Protein A/G UltraLink Resin, pre-incubated with 1-mg/ml bovine serum albumin in TBS, on a rotary shaker at 4°C overnight. Samples were washed once with IP buffer, twice with IP buffer containing 500-mM NaCl, once with wash buffer [10-mM Tris (pH 8.0), 250-mM LiCl, 1-mM EDTA, 0.5% Tergitol (Sigma) and 0.5% sodium deoxycholate] and once with TE (pH 7.5). Immunoprecipitated complexes were eluted from the resin in 100-μl elution buffer [10-mM Tris (pH 7.5), 10-mM EDTA and 1% SDS] at 65°C for 30 min. Immunoprecipitated samples and the input DNA were de-cross-linked in 0.5x elution buffer containing 0.8-mg/ml Pronase at 42°C for 2 h followed by 65°C for 6 h. DNA was purified using a polymerase chain reaction (PCR) purification kit (QIAGEN). Prior to library preparation and sequencing, the DNA fragment sizes were checked by agarose gel electrophoresis and gene-specific quantitative PCR was carried out.

### RNA isolation

RNA was extracted from exponentially growing wild-type and *Δcrp M. tuberculosis* H37Rv in Middlebrook 7H9 media, as previously described (3). Briefly, 25 ml of mid-log phase cultures were pelleted at 4°C, and RNA was isolated using the FastRNA Pro Blue kit (MP Bio), according to the manufacturer's guidelines. Following extraction, RNA was treated with Turbo DNase (Ambion) to degrade all DNA present, and the quality and integrity were assessed using a Nanodrop (ND-1000, Labtech) and Agilent bioanalyzer.

### Library construction and Illumina sequencing

For ChIP-seq, prior and post library construction, the concentration of the immunoprecipitated DNA samples was measured using the Qubit HS DNA kit (Invitrogen). Library construction and sequencing were performed using the ChIP-seq Sample Prep kit, Reagent Preparation kit and Cluster Station kit (Illumina). Samples were sequenced on an Illumina Genome Analyzer IIx (GAIIx) instrument and loaded at a concentration of 10 pM. For RNA sequencing (RNA-seq), strand-specific cDNA libraries were constructed using the Illumina Small RNA Sample preparation kit with the v1.5 small RNA (sRNA) 3′ Adaptor and sequenced on a GAIIx (Illumina).

### Data analysis

Sequence reads were aligned to the reference sequence of *M. tuberculosis* H37Rv (EMBL accession number AL123456) as single-end data using the Burrows-Wheeler aligner (BWA) (31). The genome coverage was calculated using Samtools (32) and visualized in the Artemis genome browser. For ChIP-seq, peaks were called using a combination of CisGenome (33) and the BayesPeak package in R/Bioconductor (34). For RNA-seq, differential gene expression was analysed using the DESeq package in R/Bioconductor (35). For evaluating the location of binding sites as potential transcriptional activators or repressors, a region between −200 and +20 bp with respect to the transcriptional start site was considered. For functional enrichment analysis, the 87 genes with CRP-binding sites up to 200 bp upstream from the annotated TSS (36) were analysed. All functional categories were assigned using Tuberculist annotations. GraphPad Prism v6.04 was used to compare the frequencies of different functional categories in respect to the H37Rv genome distribution using two-tailed Chi-square tests.

### Quantitative real-time PCR

Quantitative real-time PCR (qRT-PCR) was used to determine whether the ChIP experiment had worked prior to library construction and to validate both the ChIP-seq and RNA-seq data. To measure the enrichment of TF-binding targets in the immunoprecipitated DNA samples, 1 µl of IP or mock-IP DNA was used with Quantitect SYBR Green (QIAGEN) together with specific primers to the upstream region of Rv3616c, known to be bound by CRP^Mt^. To validate the RNA-seq data, specific primers were used to quantify the messenger RNA targets showing up- or downregulation, and control targets showing no differential expression. RNA was extracted as described above, and 30 ng total RNA from wild-type and *Δcrp* cells was used with the Express One-Step SYBR GreenER kit (Invitrogen) according to the manufacturer's guidelines. All primer sequences and qRT-PCR results are in Supplementary Tables S1 and S2.

### cAMP measurement

Samples for cAMP measurement during the starvation response were taken at time points 0 h, 24 h, 48 h and 96 h. At each time point, 2 ml of culture was spun down, resuspended in 0.1-M HCl and boiled for 10 min. The samples were lysed in the presence of glass beads (150–212 μm; Sigma) using a Ribolyser (Hybaid) at a speed setting of 6.0 for 40 s. The supernatant was collected by centrifugation and stored at −20°C until the assay was performed. Levels of cAMP in the cells were measured using the Direct cAMP Enzyme Immunoassay kit (Sigma), following the acetylated version according to the manufacturer's guidelines, and normalized to the total amount of protein in the samples (measured using a Nanodrop ND-1000).

## RESULTS

### Genomic mapping of the CRP^Mt^-binding profile

The aim of this work was to investigate CRP^Mt^ binding to the *M. tuberculosis* chromosome by ChIP combined with high-throughput sequencing (ChIP-seq) and to integrate these data with transcriptional profiling by RNA-seq. ChIP-seq was done using a specific antibody against CRP^Mt^, thus enabling us to study the binding under native conditions without the need to tag and overexpress the protein. Under these native conditions, CRP^Mt^ is expressed at high levels in the cell; based on published quantitative mass spectrometry and electron microscopy (36,37) and western blot analysis we estimate the number of CRP^Mt^ molecules to be approximately stoichiometric to the number of ribosomes per cell (∼3500).

**Figure 1.**
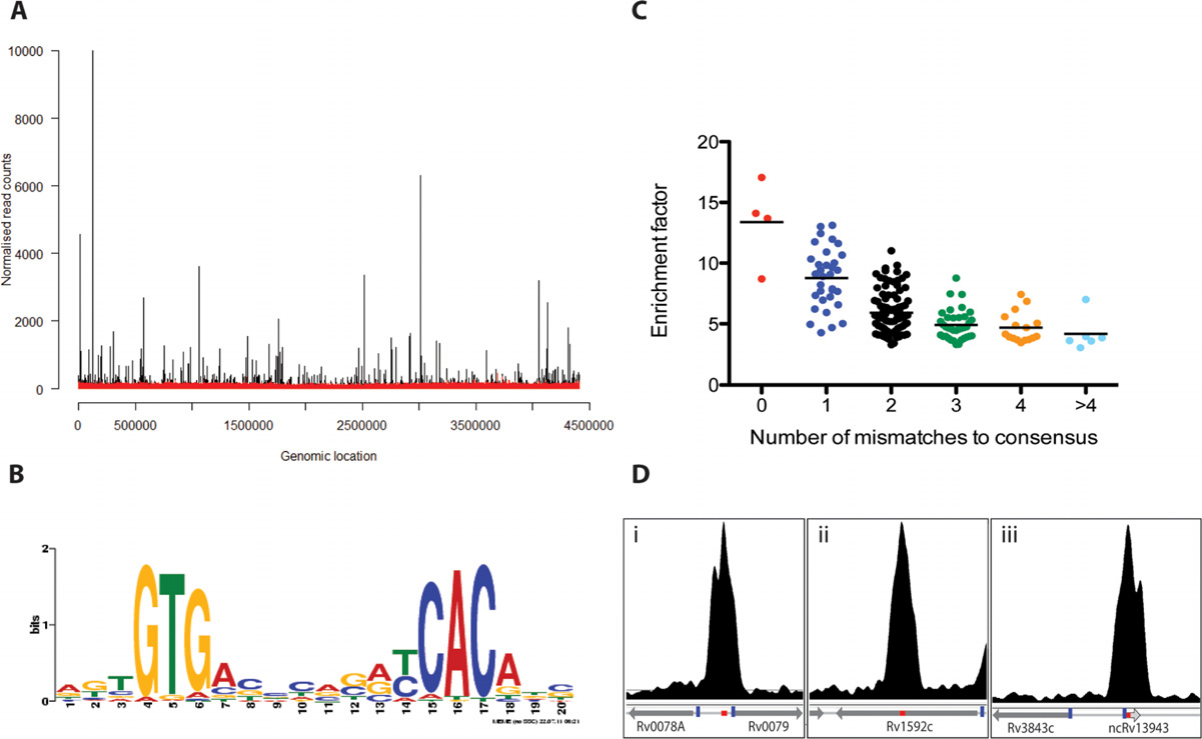
ChIP-seq mapping of CRP^Mt^-binding sites. (**A**) Sequence reads from ChIP are mapped onto the *M. tuberculosis* H37Rv genome and displayed using the Artemis browser. No peak enrichment was observed in a mock-IP sample (red trace). One hundred and ninety one peaks were identified in the IP sample using anti-CRP^Mt^ antibody (black trace). (**B**) A consensus motif resembling that defined for *E. coli* CRP was identified within 50 bp of the bound centre for 97% of the 191 of ChIP-seq peaks. (**C**) A clear correlation was observed between peak enrichment and match to the consensus motif. (**D**) Representative Artemis profiles of CBSs. CRP^Mt^ binding (i) to the divergent promoter region between Rv0078A and Rv0079; (ii) in the intragenic region within Rv1592c; and (iii) overlapping the sRNA ncRv13943. Red boxes denote the CBS and blue boxes denote the TSS.

We were able to map 98% of the sequences uniquely to the H37Rv genome (allowing for up to two mismatches per read) and achieved a near-complete representation of the entire genome (98% of the genome was mapped). The remaining 2% of the genome includes PE/PPE genes, which contain highly repetitive sequences that are poorly resolved by short-read sequencing. To visualize the genome coverage, the number of reads mapping to each position on the *M. tuberculosis* chromosome was calculated and the traces visualized in the Artemis genome browser. Peaks were called using the CisGenome software (33) to identify enriched regions in the CRP^Mt^-IP compared to the mock-IP (performed in the absence of antibody) and input (sheared genomic DNA). To validate the results, the data were also analysed using the BayesPeak package in R/Bioconductor, and peaks called in only one of the two methods were discarded. As seen in Figure 1A, there was no significant enrichment of any regions of the *M. tuberculosis* chromosome in the mock-IP (or the input; data not shown) indicating negligible non-specific binding to the resin or the antibody. In the CRP^Mt^-IP, however, 191 peaks were found, denoting CRP^Mt^-binding sites (here abbreviated to CBSs) on the chromosome (Figure 1A). The average length of the CBSs was 276 bp, and in total this represents 0.7% of the entire *M. tuberculosis* genome. No differences were observed in CRP^Mt^-DNA binding between cells grown in Dubos medium and cells grown in Middlebrook 7H9 medium (data not shown). No ChIP-seq signals were detected for the *crp*-deletion strain.

*De novo* motif discovery by MEME-ChIP (38), using 50 bp upstream and downstream of the centre of each peak, identified a consensus motif present in 97% of the 191 binding sites that is similar to motifs previously predicted for *M. tuberculosis* CRP^Mt^ and experimentally defined for *E. coli* CRP (Figure 1B). The midpoint of each ChIP peak was compared to the centre of the CRP^Mt^-binding site, as predicted from the consensus sequence, and the average difference was found to be 6 bp, with a correlation coefficient of 0.995 between the two data sets. At 14 of the sites, we identified more than one copy of the consensus motif. At three of the sites we were unable to identify a match to the consensus motif. The enrichment of the DNA fragments in the CRP^Mt^-IP compared to the mock-IP was inversely proportional to the number of mismatches found at each site (Figure 1C). The top 14 sites, with an enrichment factor (maxT) of 10 or more, are shown in Table 1 and representative Artemis profiles are illustrated in Figure 1D (for details of all sites, see Supplementary Table S3).

**Table 1.** ChIP peaks with highest fold enrichment

maxT^a^	Bound centre	CRP-binding site (CBS)^b^	ChIP to CBS (bp)^c^	Consensus mismatch	Gene	Name	CBS to TSS (bp)^d^	Location of CBS
17.1	122322	122323.5	1.5	0	Rv0104	Rv0104	+6.5	Inside 5′ end of gene
14.1	3015072	3015072.5	0.5	0	Rv2699c	Rv2699c	−62.5	between divergent genes
					Rv2700	Rv2700	−130.5	
13.7	13474	13470.5	3.5	0	Rv0010c	Rv0010c	217.5	Internal to gene
13.1	1061799	1061798.5	0.5	1	Rv0950c	Rv0950c	−69.5	between divergent genes
		1061835.5	36.5	2	Rv0951	sucC	−95.5	
					Rv0950c	Rv0950c	−106.5	
					Rv0951	sucC	−58.5	
13.0	4131452	4131461.5	9.5	1	Rv3689	Rv3689	>500	Internal to gene
12.4	2518164	2518166.5	2.5	1	Rv2245	kasA	>500	Internal to gene
12.0	1757404	1757404.5	0.5	1	Rv1552	frdA	+4.5	Upstream of operon
11.7	4057424	4057425.5	1.5	1	Rv3616c	espA	−983.5	between divergent genes
					Rv3617	ephA	−55.5	
11.6	571602	571601.5	0.5	1	Rv0482	murB	>500	Internal to gene
		571517.5	84.5	3	Rv0483	lprQ	−150.5	Upstream of gene
					Rv0482	murB	>500	Internal to gene
					Rv0483	lprQ	−234.5	Upstream of gene
11.0	2752669	2752680.5	11.5	2	Rv2451	Rv2451	>500	between convergent genes
		2752616.5	52.5	2	Rv2451	Rv2451	>500	Internal to gene
		2752593.5	77.5	2	Rv2451	Rv2451	>500	Internal to gene
10.9	2921519	2921520.5	1.5	1	Rv2591	PE_PGRS44	+16.5	Upstream of gene
10.7	2911389	2911389.5	0.5	1	Rv2584c	apt	−57.5	Upstream of gene
					Rv2585c	Rv2585c	>500	Internal to gene
10.3	1487499	1487299.5	5.5	1	Rv1324	Rv1324	+138.5	Internal to gene
		1487370.5	76.5	3	Rv1324	Rv1324	+209.5	Internal to gene
		1487064.5	229.5	1	Rv1324	Rv1324	−96.5	Upstream of gene
10.0	4329942	4329946.5	4.5	1	MT3972.1	MT3972.1	+9.5	Upstream of gene

^a^Enrichment factor (maxT) of the peaks between the CRP^Mt^-IP and the mock-IP as calculated by the CisGenome software.

^b^Centre of the CRP^Mt^-binding site based on the consensus sequence (Figure 1B).

^c^Distance of the centre of the ChIP peak (bound centre) to the centre of the CRP^Mt^-binding site.

^d^Distance of the centre of the CRP^Mt^-binding site to the TSS, as defined by (36) and documented in Supplementary Table S3.

### Location of CBSs

Sixty nine of the CRP^Mt^-binding sites mapped uniquely to a location within a protein coding gene or stable RNA, with a possible role in long-distance regulation and/or chromosome organization. Of the remaining sites, 86 CBSs mapped uniquely to intergenic loci corresponding to potential promoter regions, whilst 35 CBSs were located within a protein coding sequence in a region that could also serve as the potential promoter of a downstream gene. This represents a significant enrichment of intergenic regions over that predicted by chance, considering ∼90% of the entire *M. tuberculosis* genome is intragenic. The distribution between intragenic and intergenic locations remained approximately equal irrespective of the fold-enrichment used as cutoff, indicating that CRP^Mt^ binds with similar affinity to both types of site. In 32 cases, the CRP^Mt^-binding site was located between divergently transcribed gene pairs; this is proportional to the genome average of 16% of all genes with divergent orientation in *M. tuberculosis*. CRP regulation of divergent gene pairs has also been observed in *E. coli* (39). In some instances, CBSs mapped to the intergenic region between convergent gene pairs, like Rv2866 and Rv2867c and Rv2451 and Rv2452c. Three CRP^Mt^-binding sites also mapped upstream of the sRNAs, ncRv13843, ncRv11373 and ncRv13660c (40).

### Canonically positioned CBSs are associated with functional categories

**Figure 2.**
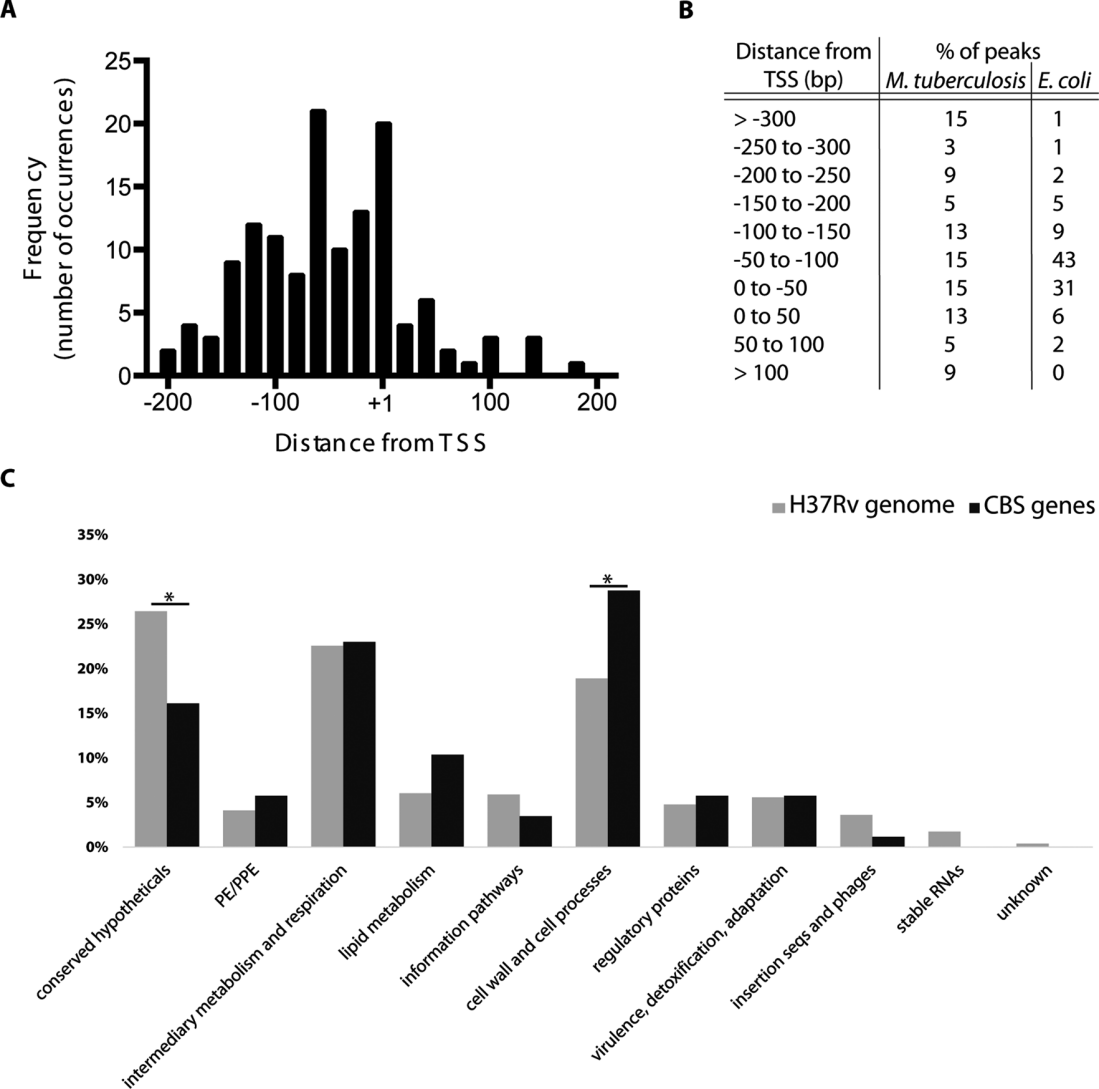
CBS distribution around transcriptional start sites. (**A**) CRP^Mt^ sites in *M. tuberculosis* show a clustering in the region around TSSs (*n* = 203). (**B**) The distribution of CBS-TSS distances for *M. tuberculosis* CRP^Mt^ sites is compared to a similar data set from *E. coli* (39). (**C**) Genes harbouring CRP^Mt^ sites in the −220 to 0 region (*n* = 87) were analysed for functional categories according to Tuberculist annotations. Bar graphs represent the percentage of each functional class for CBS genes (black bars) compared to the distribution of these classes amongst all H37Rv genes (grey bars). Asterisks denote functional categories that are statistically significant after Chi-square test analyses.

To define the precise location of CBSs with respect to transcriptional start sites (TSSs), we integrated the ChIP data set with a *M. tuberculosis* TSS map generated by sequence analysis of 5′-triphosphate-enriched RNA libraries (36). The spacing between the midpoint of each CBS motif and adjacent primary TSSs is recorded in Supplementary Table S3. Including data from peaks with multiple motifs, and CBSs mapping to more than one TSS, we measured a total of 242 CBS-TSS pairs; in 203 cases, the CBS was located within 500 bp of a TSS, 127 sites were upstream and 76 downstream, 41 of which were between the TSS and the start codon (i.e. within the 5′-Untranslated Region -UTR-) and 35 within the coding sequence. Plotting of the distribution of CBS-TSS spacing revealed clustering in the regions from −60 to −40, and from +1 to +20 (Figure 2A and B). Genes harbouring CRP-binding sites in the −200 to 0 region were analysed for functional categories. Amongst the 87 genes that contain a CRP site in a putative promoter region, genes involved in cell wall and cell processes were enriched in our data set compared to the H37Rv genome (Chi-square test, *P* = 0.021; Figure 2C).

**Figure 3.**
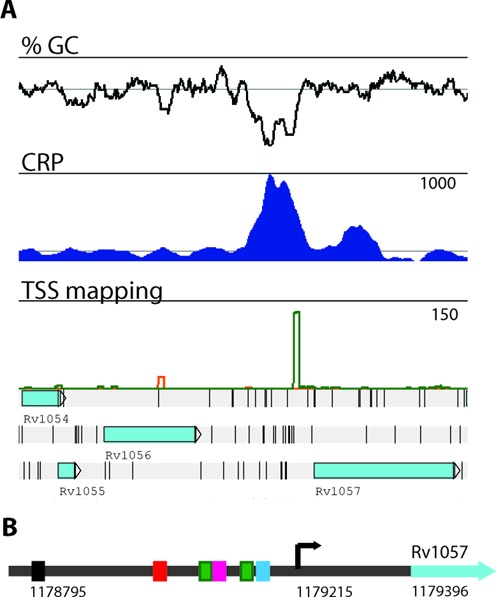
Transcription factor binding to the promoter region of Rv1057. (**A**) Artemis traces showing the binding of CRP (blue) to the AT-rich region upstream of Rv1057. TSS mapping (green) according to (36). Traces record the normalized number of mapped reads and the maximum normalized read count is indicated. (**B**) The promoter region of Rv1057 has several binding sites for other transcription factors suggesting that CRP^Mt^ may act in concert with other regulators. Transcription factor binding sites are shown as coloured boxes [black: LexA (5); red: TrcR (41); green: MprA (44); pink: EspR (7); turquoise: CRP). The arrow denotes the TSS (36). Genome coordinates indicate the start of LexA-binding site (1178795) (5), Rv1057 TSS (36) and translational start site (1179396).

Several of the CBSs have also been identified as binding sites for other transcription factors, suggesting that CRP^Mt^ may act in concert with other regulators. The promoter region of Rv1057, for example, has binding sites for MprA, EspR and TrcR in addition to CRP^Mt^ (7,41) (Figure 3). Additional promoter regions with binding sites for multiple transcription factors include *fadD26* (Rv2930) with an EspR-binding site (7) and *espA* (Rv3616c) with binding sites for EspR, MprA and CRP (7,42–45).

### Transcriptional regulation of CBS genes

Previous microarray and targeted qRT-PCR analyses have demonstrated differential expression of CBS genes following deletion of the *crp* gene (14). Using an RNA-seq approach to compare the transcriptional profile of wild-type and *crp*-deletion strains during exponential growth, we observed widespread changes in gene expression affecting more than 20% of the total transcriptome (Supplementary Table S4). Filtering based on an adjusted *P*-value of <0.05 identified 453 genes with >2-fold increased abundance in the knockout and 412 with >2-fold decrease. CBS genes comprised only a minor fraction of the differential expression profile, with statistically significant upregulation of 37 genes and downregulation of 15 genes. Forty eight per cent of the CBS-regulated genes corresponded to genes annotated as key metabolic enzymes or genes with predicted roles on transcription regulation that could amplify the CRP^Mt^ regulatory signal (Supplementary Table S5). Fifty percent of the differential expression profile (212 of the upregulated genes and 211 of the downregulated genes) was shared with the response to nutrient starvation (Supplementary Table S4), and is likely to reflect secondary effects associated with the marked growth defect of the *crp* mutant.

**Figure 4.**
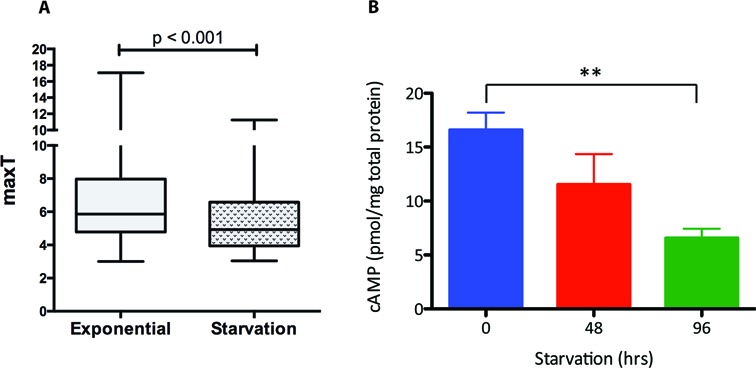
CBSs during nutrient starvation. (**A**) Enrichment of CRP^Mt^ binding to DNA during exponential and starvation phase. Box plots indicating median (horizontal line), interquartile range (box) and minimum and maximum values (whiskers) of the enrichment factor (maxT) of the 151 shared peaks between exponential growth and the starvation model (Mann–Whitney test; ** indicates significant difference between values *P* < 0.01). (**B**) cAMP concentration in the starvation model.

We anticipated that if CRP^Mt^ was acting together with other transcription factors, differential expression of CBS genes may be enhanced under alternative growth conditions. ChIP-seq analysis revealed a general decrease in CRP^Mt^ binding to DNA after incubation for 24 h in PBS (Figure 4A). Furthermore, a reduction in the amount of cAMP was observed in the nutrient starvation model (Figure 4B). There was no significant change in CRP^Mt^ protein abundance in the starvation model (36). The majority of the ChIP-seq peaks identified in exponential culture were also detected after starvation, though with a reduction in fold-enrichment and loss of 33 of the 76 peaks having an enrichment ratio of less than 5 in the exponential data set. The ChIP-seq peaks not identified after starvation were not enriched in any specific functional category and only 53% of the CBS-associated genes in exponential culture showed downregulation during starvation. Comparison of the wild-type and mutant strains under starvation conditions revealed wide-ranging differences in the overall transcript profile, with 361 genes having >2-fold higher abundance and 465 reduced abundance in the knockout (Supplementary Table S4), but again CBS genes made only a minor contribution, with 28 genes upregulated and 33 downregulated.

Twenty nine CBS genes showing a concordant response in a comparison of wild-type and *crp* deletion strains under both culture conditions are shown in Table 2, ranked according to the distance between the CBS and the TSS. Whilst the number of differentially expressed genes is low, the results are consistent with the canonical *E. coli* model of CRP^Mt^ binding close to the TSS inhibiting transcription and upstream binding enhancing transcription. There was no obvious pattern of up- or downregulation associated with CRP^Mt^ binding at sites distant from the TSS.

**Table 2.** Differential expression of CBS genes during exponential growth and nutrient starvation

			Exponential		Starved	
gene		CRP to TSS	log2 Fold Change	*P* adj	log2 Fold Change	*P* adj
Rv0046c	ino1	500	−1.22	0.000	−1.25	0.003
Rv0169	mce1A	500	0.84	0.005	2.36	0.000
Rv0469	umaA	500	−0.71	0.020	−0.99	0.043
Rv1660	pks10	500	0.87	0.036	1.02	0.044
Rv2145c	wag31	500	−0.63	0.035	−1.35	0.002
Rv2189c	Rv2189c	500	1.67	0.000	4.01	0.000
Rv2200c	ctaC	500	−2.12	0.000	−3.08	0.000
Rv3629c	Rv3629c	500	1.31	0.001	1.14	0.039
Rv0054	ssb	475.5	−1.02	0.006	−1.67	0.000
Rv3801c	fadD32	159.5	−1.37	0.000	−1.75	0.000
Rv3680	Rv3680	119.5	0.91	0.003	1.51	0.000
Rv2990c	Rv2990c	42.5	−1.89	0.000	−1.67	0.000
**Rv0104**	Rv0104	6.5	1.10	0.002	1.50	0.002
**Rv0167**	yrbE1A	3.5	0.97	0.004	2.10	0.000
**Rv1230c**	Rv1230c	3.5	0.72	0.039	1.24	0.005
**Rv0655**	mkl	−0.5	−0.88	0.002	−1.32	0.002
Rv2107	PE22	−44.5	−1.63	0.000	−2.25	0.005
Rv1592c^a^	Rv1592c	−54.5	−1.31	0.000	−1.82	0.000
Rv3219^b^	whiB1	−57.5	−1.13	0.000	−1.39	0.001
Rv1057	Rv1057	−59.5	−4.21	0.000	−2.89	0.000
Rv0452	Rv0452	−76.5	1.09	0.009	1.58	0.017
Rv0885	Rv0885	−88.5	−0.91	0.004	−1.40	0.001
Rv3053c	nrdH	−128.5	−1.41	0.000	−2.44	0.000
Rv0467	icl1	−242.5	−1.68	0.000	−2.57	0.000
Rv2173	idsA2	−246.5	1.00	0.004	1.65	0.000
Rv1379	pyrR	−365.5	1.29	0.000	1.95	0.000
Rv2703	sigA	−407.5	−1.64	0.003	−1.38	0.007
Rv2846c	efpA	−416.5	−0.93	0.001	−2.67	0.000
Rv3616c	espA	−983.5	−0.65	0.028	−1.35	0.002

CBS genes with concordant patterns of differential expression in the *crp* deletion mutant under both growth conditions are listed. According to the canonical model for CRP^Mt^ regulation, it is anticipated that genes having a CBS overlapping the transcription start site will show increased expression in the absence of CRP^Mt^ (highlighted in bold), while genes with a CBS in the upstream region will show decreased expression (underlined). CBSs between −200 and +20 bp with respect to the transcriptional start site were considered for highlighting differences in expression.

^a^additional CBS at −101.5.

^b^additional CBS at −57.5 and 47.5.

## DISCUSSION

The 191 CRP^Mt^-binding sites identified by ChIP-seq analysis have approximately equal distribution between intragenic and intergenic locations across the genome of *M. tuberculosis*. This is similar to the distribution recently reported for EspR-binding sites (7), and is intermediate between the dominant upstream preference identified by antibody-based ChIP with the ‘classical’ DosR transcription factor (4) and the predominantly intragenic distribution of NAP Lsr2 (8). Using fold-enrichment of bound sequences as a surrogate measure, there was no evidence of any difference in the affinity of CRP^Mt^ binding to intergenic as compared to intragenic sites. The antibody-based pull-down strategy we used to identify the binding profile of the native protein did not result in detection of a background pattern of low affinity sites as described for *E. coli* CRP (12), though results from high-throughput screening of tagged recombinant proteins reveal this to be a common feature of ChIP analysis of *M. tuberculosis* transcription factors (9). Whilst most transcription factors are present at low or undetectable abundance in proteome profiles measured by shotgun mass spectrometry, CRP^Mt^ resembles EspR and MtrA in having a copy number approaching that of NAPs HupB, MihF and Lsr2 (46). The number of CRP^Mt^ molecules present in the cell is in several fold excess of that required to saturate binding to the CBSs detected by ChIP-seq.

Taking advantage of a comprehensive map of *M. tuberculosis* transcriptional start sites, we were able to calculate the distance between each CRP^Mt^-binding site and the closest TSS. This revealed a pattern of clustering in upstream and downstream regions, suggesting a parallel with canonical *E. coli* CRP regulation, in which CRP binding upstream of the TSS can activate expression, and CRP binding close to the TSS is inhibitory. Data generated by combined ChIP-seq/TSS mapping reproduced previous studies of validated CRP^Mt^ regulatory targets, confirming an upstream binding site consistent with activation of *serC* and Rv0885 (13), and a binding site occluding the TSS of CRP^Mt^-repressed *frdA* (17). Alignment of ChIP-seq and TSS data sets also reproduced the more complex situation of tandem activating and inhibitory binding sites in the *whiB1* promoter (21). Additional potential internal regulatory features include identification of a CBS shared by *mmpS4*, implicated in siderophore export (47), and its potential regulator Rv0452, and CBSs upstream of both the PE13/PPE18 operon and its Rv0485 regulator (48).

Deletion of the *crp* gene had a pronounced impact on the growth of *M. tuberculosis* and on transcription profiles measured in exponential and starved cultures. The effect of *crp* deletion on expression of the set of CBS genes was similar to its effect on the overall transcriptome, with ∼20% of the genes showing increased or decreased abundance. Consistent with previous publications (13–14,17,21) our results showed that *M. tuberculosis* CRP^Mt^ can act as a ‘classical’ transcription factor in reducing or enhancing expression (dependent on spacing of CBS and TSS), but that this paradigm operates at only a subset of CRP^Mt^-binding sites. Two models can be proposed to reconcile the limited direct effect of *crp* deletion on expression of CBS genes with the extensive impact of *crp* deletion on the total transcriptome and growth phenotype. It can be envisaged that the primary transcription changes are amplified through their effect on secondary networks and co-regulation with additional transcription factors. This model is illustrated by recent mapping of the multiple regulatory layers that contribute to the overall function of the FNR transcription factor in *E. coli* (49). In an alternative model, it can be envisaged that rather than acting as primary determinants, transcription factors such as CRP^Mt^ play a complementary role within a regulatory environment dominated by global physiological control mechanisms (50). Deletion of CRP^Mt^ may have an influence on global physiology in addition to its localized effect on individual genes. CBS genes that did not show expression changes in the *crp-*deletion strain could be due to a specific role of CRP^Mt^ in regulating transcription states that will be dependent on environmental conditions or a combined regulation in conjunction with other transcription factors. This is in agreement with the findings of Hollands *et al.*, who observed no CRP-dependent regulation in *E. coli* at several promoters containing high-affinity DNA-binding sites for CRP (51). They also suggest that there may be some specific conditions where CRP-dependent regulation becomes important. Alternatively, they suggest that this may be due to CRP playing a role as an NAP, thus influencing the dynamic spatial arrangement of the chromosome (51).

There is a need for further analysis of the effects of cAMP on CRP^Mt^ binding and of the available intracellular concentration of cAMP in different growth states. In addition to its possible role in CRP^Mt^ regulation (52), cAMP binds to other transcription factors (53) and has important allosteric effects on enzyme function (54). However, the role of cAMP in CRP^Mt^ regulation remains unclear. Whilst some structural studies demonstrate a conformational change associated with binding of cAMP (55), biochemical analysis shows that this has little or no effect on binding to DNA (21). Therefore, based on the current understanding of protein allostery (56), it is likely that CRP^Mt^ is a ‘dynamic’ protein (more dynamic than *E. coli* CRP) that can readily switch to a conformation that promotes DNA interaction, even when cAMP is not bound. cAMP binding to CRP may increase the fraction of protein in the DNA-binding state, via conformational selection, which is reflected in ‘enhanced’ DNA binding. By lowering cAMP levels during starvation, the fraction of CRP in the DNA-binding conformation is lowered, thereby reducing occupancy at CRP-binding sites. This significant DNA binding by CRP^Mt^ in the absence of cAMP suggests an evolutionary adaptation given the high levels of cAMP seen in mycobacteria. Therefore CRP^Mt^ could have evolved to act as a DNA-coating protein or a recruiting protein for other transcription factors or RNA polymerase. CRP^Mt^ levels are quite high in the cell, so any decrease in cAMP levels could also be offset by the high levels of CRP^Mt^ present at any given time. However, cAMP binding to CRP resulting in the sustained occupancy of a CRP site may influence the interaction of CRP^Mt^ with other transcriptional regulators and chromosome organizing proteins.

## ACCESSION NUMBER

ChIPseq and RNAseq data have been submitted to the EBI ArrayExpress under accession number E-MTAB-2390.

## SUPPLEMENTARY DATA

Supplementary Data are available at NAR Online.

SUPPLEMENTARY DATA
