# Supplementary material for: Genomic mapping of cAMP receptor protein (CRPMt) in Mycobacterium tuberculosis: relation to transcriptional start sites and the role of CRPMt as a transcription factor
Source: Nucleic Acids Res. 2014 Jun 21;42(13):8320–9. doi: 10.1093/nar/gku548 (PMC4117774; doi:10.1093/nar/gku548)
Supplement: SUPPLEMENTARY DATA [file supp_42_13_8320__index.html]

Genomic mapping of cAMP receptor protein (CRPMt) in Mycobacterium tuberculosis: relation to transcriptional start sites and the role of CRPMt as a transcription factor — SUPPLEMENTARY DATA 

# Genomic mapping of cAMP receptor protein (CRPMt) in *Mycobacterium tuberculosis*: relation to transcriptional start sites and the role of CRPMt as a transcription factor

## SUPPLEMENTARY DATA

**Files in this Data Supplement:**

- SUPPLEMENTARY DATA
- SUPPLEMENTARY DATA
