# Supplementary material for: Genomic mapping of cAMP receptor protein (CRPMt) in Mycobacterium tuberculosis: relation to transcriptional start sites and the role of CRPMt as a transcription factor
Source: Nucleic Acids Res. 2014 Jun 21;42(13):8320–9. doi: 10.1093/nar/gku548 (PMC4117774; doi:10.1093/nar/gku548)

Supplementary Data

Genomic mapping of cAMP receptor protein (CRP^{Mt}) in *Mycobacterium tuberculosis*: relation to transcriptional start sites and role of CRP^{Mt} as a transcription factor

Christina Kahramanoglou[1], Teresa Cortes[1*], Nishad Matange[2], Debbie M. Hunt[1], Sandhya S. Visweswariah[2], Douglas B. Young[1,3], Roger S. Buxton[1]

Supplementary Table S1. Primers used for RNA-seq validation through real-time quantitative PCR analyses

RNA-seq CRP validation primers		
Rv Number	Forward sequence	Reverse sequence
Rv2989	CATAGCGGTATCGGTGTCCT	CAGTGGATCGTCGACATGG
Rv2987c	CCTTTCACACCCACTCTGGT	GGGACTGAGGTTTAGCACGA
Rv2988c	GGACACCGCACTCGTCTACT	GAAGGTCCATGTACGCCAAT
Rv3229c	ACCAAATCGAGCATCACCTC	ATCGTCAGCGTTGTCACGTA
Rv3093c	CGATATCGAAGTCGCCCTAC	AGAGAAGCGACCGAACTGAC
Rv3676	ATCTCGGGGAAGGTCAAGAT	CAGCAGCTGTTCGGAGATTT
Rv1622c	CCGGACTGTTCTCGCTCTAC	GTCTTTGCCGTATGCCAGTT
Rv1621c	CGTCGATTCAGTGGCTACAA	GGCAAATAGCCGGTGAAGTA
Rv1620c	ACGAACCTGTCGAACACCTC	TGTTGTTGTCGCTGGAGTTC
Rv2699c	TTGAAGAGCTCAAAGCACGA	AGCAACTCGAGCAGGTGAAT
Rv3290c	ACGAGTTTGATGCACTGCTG	GTAAGATTGCCACCCCATGT
Rv3289c	GGGGTCGGTTTCCTCATTAT	TCAGCGTCAACAAAAACCAG
Rv1738	GATCGACGAACACGAAGGAT	GGGTCGACACCTTCAACATT
Rv0192	GCCCAAGGCTACAAGTTGAC	GCCGAAGTTGTCGTAGAACC
Rv3299c	TACGACCCGATCATCAGTCA	GTCGCGTAGTACAGCATCCA

Supplementary Table S2. qRT-PCR

	qPCR		RNAseq	
Rv Number	LogFC	adjPvalue[1]	LogFC	adjPvalue[2]
Rv0192	-1.82	0.0307	-0.008	1
Rv1620c	2.65	0.0004	5.147	0.064004603
Rv1621c	1.364	0.8378	4.129	0.321309088
Rv1622c	1.58	0.1333	3.804	0.327562536
Rv1738	2.12	0.0089	6.542	0.138588581
Rv2699c	1.93	0.0242	4.235	0.138588581
Rv2987c	-7.87	< 0.0001	-5.737	0.020729412
Rv2988c	-10.218	< 0.0001	-5.225	0.020729412
Rv2989	-2.189	0.0002	-5.79	0.116960149
Rv3093c	-5.094	< 0.0001	-3.718	0.359847866
Rv3229c	-6.757	< 0.0001	-4.07	0.144696504
Rv3289c	2.08	0.9997	4.923	0.138588581
Rv3290c	3.973	< 0.0001	4.316	0.124462679
Rv3299c	-1.351	0.0224	-0.003	1
Rv3676	-13.635	< 0.0001	-8.344	0.001401907

[1]Adjusted pvalues for qRT-PCR data calculated using Sidak’s multiple comparisons test.
[2]Differential expression analysis for RNAseq data was performed using the DESeq package.

Supplementary Tables S3, S4 and S5.

Provided as a separate excel file

Supplementary Figure S1. Western blot showing specificity of specific α-CRP^{Mt} antibody. WT H37Rv *M. tuberculosis* cultures were grown to mid-log phase. A serial dilution of the cell lysate was analysed by SDS-PAGE (lanes 1-4 lowest to highest dilution) and western blot analysis using specific α-CRP^{Mt} antibody.

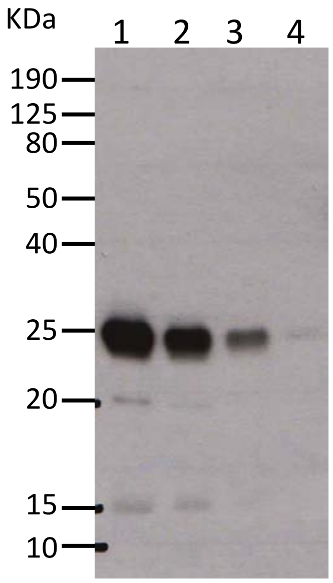

Supplement: SUPPLEMENTARY DATA [file supp_gku548_nar-00899-x-2014-File006.pdf]
